# Supplementary material for: Low versus high dose anticoagulation in patients with Coronavirus 2019 pneumonia at the time of admission to critical care units: A multicenter retrospective cohort study in the Beaumont healthcare system
Source: PLoS One. 2022 Mar 24;17(3):e0265966. doi: 10.1371/journal.pone.0265966 (PMC8947132; doi:10.1371/journal.pone.0265966)
Supplement: S1 Data — (DOCX) [file pone.0265966.s001.docx]

Coding of the variables included in the database:

1. Age: Age of patients in years
2. Gender: 0-Male, 1-Female
3. Race: 1-White, 2-Black, 3-Asian, 4-Indian or Alaskan, 5-Other
4. BMI: Body mass index.
5. AC_Highdose: 0-Low dose anticoagulation, 1-High dose anticoagulation
6. Thrombosis: 0-No, 1-Yes
7. Bleeding: 0-No, 1-Yes
8. Intubation: 0-No, 1-Yes
9. DurationOfIntubation: Duration of intubation in days
10. LOSinICU: Length of stay in the ICU/SDU in days
11. Mortality: 0-No, 1-Yes
12. Fibrinogen: level on admission to the hospital
13. Ferritin: Level on admission to the hospital
14. DDimeronICUadmission: D-Dimer level on admission to ICU/SDU
15. Paduascore: Padua score
16. mSOFA: modified SOFA score
17. HTN: History of hypertension; 0-No, 1-Yes
18. DM: History of diabetes mellitus; 0-No, 1-Yes
19. Asthma: History of asthma; 0-No, 1-Yes
20. Afib: History of atrial fibrillation; 0-No, 1-Yes
21. HF: History of heart failure; 0-No, 1-Yes
22. COPD: History of chronic obstructive pulmonary disease; 0-No, 1-Yes
23. Cancer: History of cancer of any type; 0-No, 1-Yes
24. CAD: History of Coronary artery disease; 0-No, 1-Yes
25. TIA_Stroke: History of transient ischemic attack or stroke; 0-No, 1-Yes
26. CKD: History of chronic kidney disease; 0-No, 1-Yes
27. VTE: History of venous thromboembolism; 0-No, 1-Yes
28. Other: Other comorbidities; 0-No, 1-Yes
29. NumberOfComorbidities: Total number of comorbidities
