# Supplementary material for: Low versus high dose anticoagulation in patients with Coronavirus 2019 pneumonia at the time of admission to critical care units: A multicenter retrospective cohort study in the Beaumont healthcare system
Source: PLoS One. 2022 Mar 24;17(3):e0265966. doi: 10.1371/journal.pone.0265966 (PMC8947132; doi:10.1371/journal.pone.0265966)
Supplement: Attachment — Submitted filename: Response to Reviewers 1.16.docx [file pone.0265966.s003.docx]

Review Comments to the Author

Reviewer #1:

Comment: The authors conducted a study of treatment with high dose anticoagulation at the time of ICU/SDU admission which was associated with decreased adjusted mortality among critically ill adult patients with COVID-19 infection. The major concern of this retrospective study is how to decide the dosage of anticoagulation. Since the D-dimer and fibrinogen levels were similar between the two groups, how the doctors used higher or lower dosage of anticoagulation?

Answer: Thank you for highlighting this important question. This study utilized retrospective data at the beginning of the pandemic. There was only little known about the effect of anticoagulation on Covid-19 outcomes. Therefore, the decision of anticoagulation dosage was based entirely on the treating physician preference in a near random fashion. Indeed, this was one of the points that encouraged us to conduct this study, as less bias would be anticipated. The manuscript is updated to reflect this point.

Reviewer #2:

Comment: How many of these patients were on vasopressors, CRRT, how many required NIV support and how many were on high flow 0_2_, although a mention was made about the intubated patients. Was there any correlation between these and mortality?

Response: We agree with the reviewer on the potential effect of these factors on the investigated outcomes. These variables were accounted for through the mSOFA score, as a composite measure of end organ damage. We did not include these variables as individual variables. The admission criteria to our ICU/SDU units at that time included patients on high flow, NIV, or intubated, and divided the population into intubated and non-intubated (both High flow and NIV populations). It was felt that the sample size will be very small for these populations (NIV and High flow patients individually) and any conclusions drawn on these populations will be under powered, therefore they were included as one group.

Comment: Was there any association between x ray pattern, CT severity score and D dimer levels?

Answer: We did not study the imaging findings for these patients. D-dimer level was investigated, but there was no association between its level and inpatient mortality, thus it was not included in the multivariable analysis. In addition, we performed a subgroup analysis based on the D-dimer level.

Comment: Was a bed side 2 D echo done and if so how many of them had PAH or RV dysfunction?

Answer: Unfortunately, we did not collect the echocardiography studies results for these patients. We do not believe a significant number of patients had echocardiography done at that time due to lack of PPE, unless absolutely necessary.

Comment: Was there sepsis induced coagulopathy. Were procalcitonin levels done in this study? Possible association between development of antiphospholipid antibodies, notably lupus anticoagulant (LAC) has been seen in many studies which may contribute to coagulopathy, was there any such observation in this study?

Answer: Coagulopathy, as part of mSOFA score, was included in the study but not a separate variable. No data were collected regarding procalcitonin or lupus anticoagulant was investigated in the study.

Comment: How many of these patients received steroids, remdesivir? There has been a weak association between the treatment modalities used in COVID especially more so with corticosteroids resulting in a thrombogenic state.

Answer: Thank you for highlighting this important potential interaction. During the time of conducting this study, neither the Remdesivir nor the steroids were approved for treatment of Covid-19, therefore none of our patient were treated with them and we can’t explore such interactions. The manuscript was updated to reflect these limitations.
